# Supplementary figures and images for: The Endocrine Disruptor Compound Bisphenol-A (BPA) Regulates the Intra-Tumoral Immune Microenvironment and Increases Lung Metastasis in an Experimental Model of Breast Cancer
Source: Int J Mol Sci. 2022 Feb 25;23(5):2523. doi: 10.3390/ijms23052523 (PMC8909997; doi:10.3390/ijms23052523)

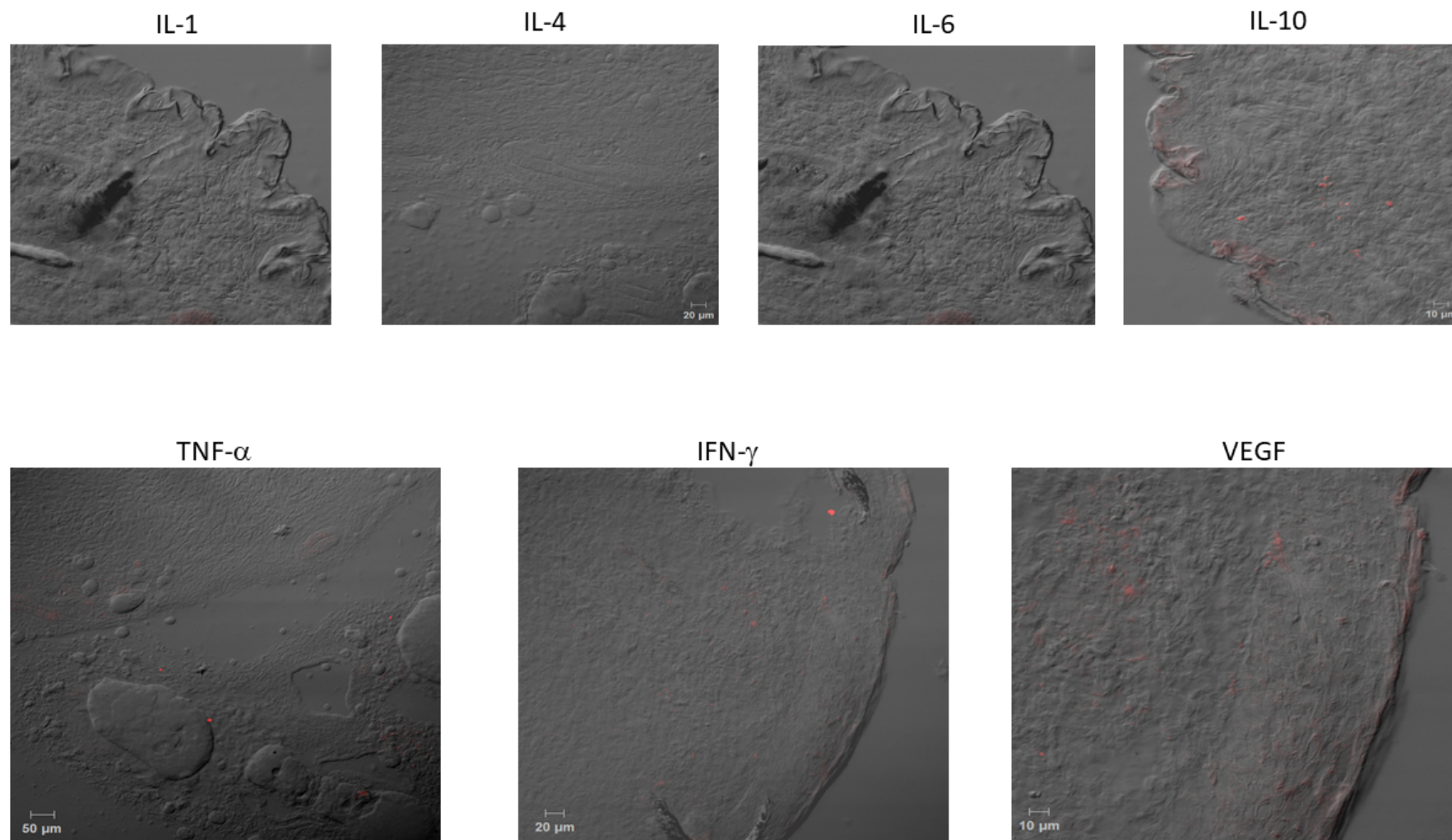

Figure S1. Different negative controls of each cytokine evaluated.

Supplement: Supplementary file 1 [file ijms-23-02523-s001.zip › ijms-1596612-supplementary.pdf]
